# The relevance and role of homestays in medical education: a scoping study

**DOI:** 10.1080/10872981.2017.1320185

**Published:** 2017-05-02

**Authors:** Bonnie Olivia Hughes, Mosa Moshabela, Jenni Owen, Bernhard Gaede

**Affiliations:** ^a^ Centre for Rural Health, University of KwaZulu-Natal, Durban, South Africa; ^b^ Discipline of Rural Health, University of KwaZulu-Natal, Durban, South Africa; ^c^ Center for Child and Family Policy, Duke University, Durham, North Carolina, USA; ^d^ Discipline of Family Medicine, University of KwaZulu-Natal, Durban, South Africa

**Keywords:** Community-based education, cultural exchange, homestay, student accommodation, undergraduate medical education

## Abstract

**Background**: The community-based medical education curriculum is growing in popularity as a strategy to bring universal health coverage to underserved communities by providing medical students with hands-on training in primary health care. Accommodation and immersion of medical students within the community will become increasingly important to the success of community-based curricula. In the context of tourism, homestays, where local families host guests, have shown to provide an immersive accommodation experience.

**Objective:** By exploring homestays in the educational context, this scoping study investigates their role in providing an immersive pedagogical experience for medical students.

**Design**: A scoping review was performed using the online databases ScienceDirect and the Duke University Library Database, which searches Academic Search Complete, JSTOR, LexisNexis Academic, Web of Science, Proquest, PubMed and WorldCat. Using the inclusion term ‘homestays’ and excluding the term ‘tourism’, 181 results were returned. AClose assessment using inclusion criteria narrowed this to 14 relevant articles.

**Results**: There is very little published research specific to the experience of medical students in community homestays, indicating a gap in the literature. However, the existing educational outcomes suggest homestays may have the potential to serve a significant role in medical education, especially as a component of decentralised or community-based programmes. The literature reveals that educational homestays influence language learning, cultural immersion, and the development of professional skills for health science careers. These outcomes relate to the level of engagement between students and hosts, including the catalytic role of community liaisons.

**Conclusions**: Homestays offer a unique depth of experience that has the potential to enrich the education of participating students, and require further research, particularly in the context of distributed and decentralised training platforms for medical and health sciences students. Future studies should explore the potential for homestays as a pedagogical component of community-based medical curriculum.

**Abbreviations:** CBME: Community-based medical education

## Background

The World Health Organization (WHO) called for a worldwide shift towards primary healthcare and a focus on community health in order to achieve global health equity by 2000, yet many areas of the world remain underserved today [1]. Medical schools recognize their crucial role in training physicians to better reach and serve these locations. As a result, many have developed community-based education (CBE) medical curriculum [2,3]. Community-based education is described as ‘learning activities that use the community extensively as a learning environment’ which, when applied in the medical context, relies on the depth of engagement of medical students, teachers, and community members [4]. The CBE approach to medical training – community-based medical education, or CBME – exposes medical students to the realities of healthcare in underserved communities, which has shown to improve their abilities to serve in these communities while also increasing empathy and communication skills [2,5]. Simultaneously, literature shows CBME benefits the participating communities by increasing awareness of health behaviours and disease prevention while also improving relationships between underserved communities, their hospitals, and the medical universities [3]. CBME programmes typically engage students with the local environment through activities like home visits, mobile clinics, and health workshops [3]. This study examines the role of homestays, where students live with a local family, as a tool for CBME. For this study, homestays are described as accommodation in a local home that includes interaction with the family and inclusion of the student guest as a member of the family. For example, homestay host families typically include the student guest in mealtimes and social events. It is important to note that although basically, a homestay serves as accommodation, we are focusing on the environment and opportunities it creates for learning among those involved. This environment is highlighted by tourism literature, which declares homestays as a popular choice among tourists because they offer unique access to an authentic cultural experience, providing ‘emotional value’ [6–8]. Just as they serve tourists, homestays may potentially serve as platforms for immersion of medical students in the community setting. Using a scoping review methodology, this study explores the existing literature related to homestays for medical students to investigate how the model affects educational experiences. This review presents the current state of evidence with regards to the outcomes of homestays, and also highlights research gaps related to medical education. The review argues that homestays may play an important role in shaping medical curricula, especially with the recent growth of decentralised and distributed community-based medical education programmes.

## Methods

### Purpose of the scoping study

A scoping study is useful in mapping the range of literature that exists around a topic of interest and helps to focus the research questions by charting existing research findings and identifying research gaps [9,10]. The methodology is also considered a useful approach for determining the necessity and value of a full systematic review, and should not be treated as a systematic review [9]. A scoping study best suited this topic since little is known about homestays, making a literature mapping exercise necessary. Notably, the approach allows for the inclusion of a variety of study designs and follows a systematic process to documenting, charting and analysing the outcomes. In this case, the scoping study provided insight into what gaps exist in the study of homestays for CBME.

### Selection of the included literature

After a comprehensive review of the existing literature, the authors found that the majority of literature relevant to homestays was in both tourism and educational contexts. We explored the educational homestay literature since this study focuses on homestays as a component of community-based medical education. The study included searches of two online databases: ScienceDirect and the Duke University Library Journal Database (which searches Academic Search Complete, JSTOR, LexisNexis Academic, Web of Science, Proquest, PubMed and WorldCat). These databases were chosen because of their wide scope of coverage and only journal articles were included. In order to narrow the returns, ‘homestay’ was searched, excluding ‘tourism’. There was an initial attempt to search ‘homestay’ and ‘education’ but it yielded insufficient results. A final search in October 2015 for ‘homestay’ excluding ‘tourism’ returned 181 results, 103 from ScienceDirect and 78 from the Duke University Library. Date restrictions were not set in order to broaden the search, so as to capture the full scope of the homestays literature. After a title review and the elimination of duplicates, 118 articles were excluded. Another 28 publications were eliminated after the abstract review, and a final full article review excluded 21 more articles. For the final article review, articles were included if they met the following criteria: Article describes/analyses the use of a homestay as accommodation, is not focusing on/analysing a homestay used for tourism purposes, and the homestay is used in an educational context where the guests are described as students.

The final selected literature included fourteen publications. Nine qualitative studies, one quantitative, and four mixed method studies were included. This variety of study designs is one of the benefits of using a scoping study methodology. Each of these publications were analysed for their quality using a series of assessment questions, which are outlined in Table 1. These steps were taken to mirror the rigor of a search method often used in a systematic review while still achieving the goals of a scoping study.

**Table 1.** Quality assessment.

Study type	Methodological quality criteria
All study designs	Are there clear research questions/objectives?
	Do the data collected allow research questions to be appropriately addressed?
Qualitative studies	Are the sources of data relevant to address the research question?
	Is the process for analyzing data relevant to address the research question?
	Is appropriate consideration given to how findings relate to the context, e.g. the setting in which the data were collected?
	Is appropriate consideration given to how findings relate to the researcher’s influence, e.g. through their interactions with participants?
Quantitative studies	Is the sampling strategy relevant to address the research questions?
	Is the sample representative of the population under the study?
	Are measurements appropriate (clear origin or validity known or standard instrument)?
	Is there an acceptable response rate (60% or more)?
Mixed methods	Is the mixed methods design relevant to address the qualitative and quantitative research questions?
	Is the integration of qualitative and quantitative data relevant to address the research questions?
	Is appropriate consideration given to the limitations associated with this integration, e.g. the divergence of quantitative and qualitative data in a triangulation design?

### Charting the results

The selected literature was read closely for the extraction of methods, educational outcomes, and homestay recommendations. Each publication was studied to ensure the homestay was represented in an educational context. Table 2 describes the ten questions that were answered and charted for each study. The charted outcomes were then analysed for patterns across the literature. Themes that emerged from the outcomes included language learning, cultural immersion, professional skills, negative experiences as well as recommendations that fell into training, community liaison placement, communication, and evaluation. A secondary chart groups similar results into the emerging thematic categories in order to assist in organizing the review.

**Table 2.** Charting of included articles.

1	Date of publication
2	Location of study
3	Study population – must be students or homestay participants (i.e. hosts)
4	Study methods – what tools were used to collect data or assess educational outcomes
5	Study design – qualitative, quantitative, mixed methods
6	Length of the homestay experience being evaluated
7	Demographics of the students and hosts (i.e. are the students living in a foreign country)
8	Outcomes of the homestay experience
9	Recommendations from the researchers
10	Relevance to the educational value of homestays

## Results

Fourteen studies explore homestays in the educational context, indicating the limited amount of research into this topic. Of these fourteen studies, four were mixed methods, nine qualitative, and one quantitative. This diversity is one benefit of using a scoping study methodology. These studies also occur in diverse locations, including Canada, Ecuador, Thailand, Nicaragua, Mexico, China, Russia, England, Spain, Tunisia, Luxembourg, and the United States. Therefore, homestays have international implications and can occur in a variety of settings. Only three of the resulting papers were specific to medical education, highlighting a definite knowledge gap in the role of homestays in this context. As a result, this study draws from the outcomes of diverse educational homestay programmes in order to assess their relevance to medical education. Across the selected literature, the homestay process is described and common outcomes are investigated. This study first examines the structure, design, process, and definition of homestays as described by the included literature. This is followed by homestay outcomes including language learning, cultural immersion, and professional skills. Finally, the research gaps and need for further research in this area are discussed.

## Homestay characteristics

Homestays are not standardized and occur in diverse settings. The included studies describe homestays in diverse ways: some are single-parent homes, some two-parent, and many have children, but they vary in age. Students are sometimes hosted in the home individually while other times they are hosted in pairs. Some programmes include financial support for the host family to share food with the student while others just require that the student be provided with access to the kitchen. Homestays are typically offered as an alternative to student accommodation in, and therefore differ by design from, dormitories or independent apartments. The length of stay reported by these studies varied from three weeks up to two years. Despite the diversity across programmes, there are consistent recommendations in the literature on how to properly design, prepare, and maintain a homestay programme for students.

Training or pre-departure orientation for hosts and students is recommended by the majority of the included literature. One study found that students felt unprepared for their experience abroad, resulting in negative outcomes and perceptions of the programme [11]. In this case, the students came from an urban environment to live in a rural setting and were not aware of the lifestyle differences and found it difficult to adjust [11]. Preparing students could have prevented this negative experience. Students and hosts should also be informed on what to expect in terms of cultural and lifestyle differences they may encounter in order to avoid a misalignment of expectations [12]. This is informed by the experience of participants in the Kobayashi and Viswat study [13], who reported conflict surrounding expectations of the student-host relationship. Preparation of students and hosts can also improve skill development to help deal with conflicts that may arise. Research suggest that communication can be crucial in the solving of conflict in a homestay situation [13,14]. Focusing on communication skills during preparation makes it more likely that the relationships in the home will flourish and serve as a positive experience [12,15]. Cultural sensitivity training is also likely to help participants adjust in a new environment and to potentially increase empathy [16]. Another important focus of pre-departure can be on how to engage and interact, which has shown to influence the outcomes of a homestay [12,15,17]. This includes encouraging hosts and students to interact, form meaningful relationships, and actively try to gain deeper knowledge about the surrounding language and culture.

Another suggestion for running a successful homestay programme is to place an individual on site in the role of community liaison who can assist with homestay management and conflict resolution. Having this person in place can help students engage in successful relationships as mentioned above. The main complaint of high school students who struggled to adjust in the Canadian homestays was that when they had a concern, it was difficult to get help from programme management, leaving them feeling unsupported [11]. Placing someone on site creates an accessible point of communication for both students and hosts so that concerns are addressed as soon as possible. This set-up creates a reliable, efficient programme that is more likely to succeed over time. One of the studied health science programmes makes use of a third party person who is local to the community, which has shown to help build legitimacy and strong networks between the programme participants and host community [18]. This person can also help student integration into the community and can bridge cultural gaps to aid with immersion [19].

## Homestay outcomes

With the establishment of homestay programmes for students, certain educational outcomes are reported.

### Language learning

Four of the selected studies focus specifically on language development through a homestay experience.

Homestays are commonly used as accommodation for students in study-abroad situations. When the student stays in a country where a different language is spoken, the ‘homestay advantage’ claims that living with a local family will increase language proficiency more than other accommodation options, like a student dormitory or independent apartment [17]. Research suggests that living with a family provides more consistent language immersion, which ultimately leads to a deeper level of learning and use. A study of Korean college students in American homestays tested English reading, writing, speaking, and listening skills before and after their stay to verify the homestay advantage [20]. Overall, student English proficiency did not significantly improve, but a minor increase in speaking fluency was seen. These study participants only spent three weeks in English-speaking homestays, which authors suggest may not have been enough time to significantly affect proficiency [20]. Two studies of language development seem to confirm the homestay advantage by reporting significant oral proficiency increases for university students who lived in homestays while abroad [15,21]. One of these studies looked at the individual change in language performance before and after the experience and found that students who reported a positive homestay experience were likely to have improved speaking skills [15]. These students reported high levels of interaction with host families, indicating that language proficiency and the homestay advantage may be dependent on this type of engagement between students and hosts [15]. The second study not only calculated individual student results, but compared these to results of students in programmes that lacked the homestay component [21]. The students who stayed in homestays tested significantly higher than students who studied with a different accommodation situation [21]. Homestay students also reported a high level of confidence in their language gains and felt comfortable with local social interactions [21]. However, the homestay advantage is called into question by Rivers [17] who tested speaking, reading, and listening language skills and found a decrease in oral proficiency of homestay students compared to dormitory students. This was attributed to the fact that students reported spending very little time actually speaking or engaging with their host families [17]. This shows how educational outcomes may be linked to level of immersion. Table 3 describes the results of each study that investigated language development of students in a homestay.

**Table 3.** Comparison of language proficiency results.

	Comparative	Speaking	Reading	Writing	Listening
Byun and Kim 2013 [20]	Individual post-test compared topre test	Minor increase	Insignificant increase	Insignificant decrease	Insignificant increase
Rivers 1998 [17]	Change from pr- to post-test compared todorm-stay students	Significant decrease	Significant increase	n/a	Insignificant result
DiSilvio et al. 2014 [15]	Individual post-test compared topre-test	Significant increase	n/a	n/a	n/a
Shiri 2015 [21]	Change from pre- to post-test compared to results of otherArabic learning students	Significant increase	n/a	n/a	n/a

### Cultural immersion

Many study abroad homestays not only serve a language learning purpose, but also provide access to a unique cultural experience. A study of why certain students chose to continue language study beyond the required curriculum found that interactions and relationships with native speakers encouraged this continued learning [22]. Students report that homestays can be a first step to building local relationships. They also reported developing a passion for the culture in which they studied, making them want to become even more immersed [22]. In their 2014 study, Rodriguez and Chornet-Roses [12] identify four types of relationships that emerged in a student homestay: family, friend, guest-host, and tenant-landlord. The family type relationship was described as a result of close bonds formed, participation in family activities and generally ‘feeling at home’. The friend relationship was more surface level where some quality time was spent together but the feeling of a family bond was not developed. The guest-host relationship formed where the student felt it was important to avoid imposing on the host, and the situation felt more like a cohabitation. The tenant-landlord level relationship was characterized by isolated student living arrangements and a business-like relationship with the hosts. This diversity in relationships reflects the scale of depth of immersion possible within a homestay – if a family-type relationship is established, the guest is more likely immersed in the local culture, while a tenant-type guest may be more of a cultural observer [12].

According to the study of a language intensive programme in Tunisia, 77% of participants strongly agreed that the homestay experience made them feel better connected to the community, showing the benefits of such a programme even beyond the home [21]. These students interacted with broad social networks within the community and 85% reported the homestay as offering valuable insight into the local culture [21]. Homestays provided students with a connection to community members they otherwise may not have had, which has strong implications in community-based educational programming.

In some of the studied homestay programmes, cultural differences were an obstacle to immersion but resulted in the development of cultural competency. Host mothers reported concerns with certain student behaviour, yet they attributed many of the issues they confronted to a cultural mismatch, and downplayed them as problems [23]. For example, there were many cultural differences reported by hosts surrounding the issue of food etiquette and behaviour [23]. These cultural differences have shown to be good learning experiences where both hosts and students work to communicate and find solutions. Engaging in these discussions has potential to deepen the relationship between host and student [12]. Hosts also reported less conflict with students who stayed a longer period of time, reflecting how a relationship builds between the student and host, making it easier to communicate and handle differences [23]. Jackson [16] studied the change in cultural sensitivity of university students over a homestay experience. Using the Developmental Model of Intercultural Sensitivity, it was found that most students generally started in the sensitivity stages defined as denial, defence, or minimization. In these stages, students tend to judge the host culture based on comparisons to their own [16]. The students who progressed into the high sensitivity stages of acceptance and adaptation recognized cultural differences and accepted them as part of the experience [16]. The highly sensitive students also showed increased empathy, an important component of cultural competency [16]. Though these outcomes were from the study of general students, they emphasize the role homestays can have in the development of cultural competency, which is an important component of medical student training.

### Professional skill development

Though there were few resulting studies examining homestays in a specifically medical student context, three papers did explore community-based medical curriculum. Rassiwala et al. [19] compared two programmes: one model a week-long, clinically intensive medical training experience and the other, a month-long immersive experience where students lived in homestays. Though the first model was successful in advancing clinical skills, the second model allowed students more observation and interaction time, leading to a better understanding of community health. Homestays allowed these students the level of immersion that made it possible for them to more fully understand the complexities of lifestyle and disease and the challenges of global health [19]. A second study of health science field courses found that 91% of students who lived in homestays while serving the community report using cross-cultural skills in their health career while 63% use their language skills, which are both largely attributed to the immersive living experience [18]. Health science students also report that they believe an immersive experience should be a required part of their education, showing their support for these programmes [18]. One survey of health science students who had a global immersive experience has collected reports ten-years post-programme, and found that students strongly believe this experience ultimately effected their career decisions [24]. This shows the influence immersive community-based medical programmes can have on the future of physicians and ultimately, health care.

## Discussion

Homestays have shown to be an enriching experience for students and should be explored further in the context of community-based programmes for medical students. It is clear throughout the literature that the priority must be to meet student accommodation needs, as when students are not comfortable living in the home or feel unsupported, it is difficult for the experience to be positive [11,13]. Researchers recommend a hands-on approach to management by placing someone on site to control homestay communications and solve conflicts efficiently, as well as to conduct consistent evaluations and make necessary changes [11,15,19]. Once a functional accommodation programme is created that meets host and student needs, the homestay can create a valuable environment for interaction and learning. However, research shows that learning outcomes are dependent on the engagement between the student and host [15,17,22]. Especially in language learning, without high levels of engagement and interaction students cannot expect that living in a homestay will increase their foreign language proficiency [17,20]. Hosts also play a role in engaging with their student and can take an active role in the students’ learning [23]. Preparing students and hosts before the experience can encourage the necessary actions to lead to the educational opportunities a homestay has to offer [11,15]. When students do engage with their hosts, they become more integrated into the family and have closer access to cultural experiences, depending on the depth of their relationship [12]. Relationships start within the home and can then extend into the host community, creating opportunities for students beyond what they would have in a different accommodation situation [12,21,22]. Valuable skill development can result from the engagement and immersion offered by a homestay.

Ultimately, this study exposes a large knowledge gap on homestays in the medical education context. However, the outcomes of homestays for general students are concrete and likely to translate to the medical student context. Since community-based and decentralised or distributed learning curriculum is a growing component of medical education, accommodation can be difficult to provide and maintain in these settings. The depth and diversity of experience offered by homestays provides accommodation while also contributing to the learning objectives of these programmes. Homestays can deliberately offer medical students the exposure to authentic lifestyle, while intentionally welcoming community members to interact with these students beyond the clinical setting. This connection with the community is beneficial for health science students, as it helps them develop cultural competence skills and a holistic understanding of health [18,19,24]. The communication skills developed living in a homestay are also extremely important for health science students, as communication is crucial in the medical field. Participating communities also may experience positive outcomes from hosting medical students, especially in rural or disadvantage locations, which are commonly the setting for CBME [3,5,25]. Tourism homestay literature reports outcomes including economic benefits, business skill development, and community empowerment [25,26]. Changing medical curriculum to include community-based education may bring these outcomes to communities that may not attract tourism. Ultimately, this is predicted to improve the quality of care in otherwise neglected areas while also increasing the recruitment of health care workers to places in need [5]. These outcomes illustrate the significant role homestays can play in medical education, not just as accommodation but as an actual element of the curriculum. The literature suggests this is an area worth further exploration as decentralised medical training expands.

### Limitations

The scoping study methodology may limit results due to focus on mapping studies, rather than assessing validity of the research conducted. It is also possible that research on homestays exists under different terminology or language, not considered in this study. Within the selected literature, there is a strong focus on homestay outcomes and less discussion on the logistics of the homestays, which would likely vary depending on characteristics of the homestay contexts. These deficiencies open up knowledge gaps in the details of the host family makeup and the number of students per home, which are factors that likely influence the homestay experience. Since so few studies examine homestays for medical students, we draw conclusions from other contexts, and it is possible these outcomes would not translate to the medical context. However, these lessons indicate the need for further research on homestays in the context of medical education, so that concrete conclusions on the outcomes of homestays can be made as applicable to medical students across the world.

## Conclusions

Educational homestay research shows the model serves as viable accommodation for exchange students while also enriching their educational experience through cultural immersion and relationship building. Adding a homestay component to community-based and decentralised medical education programmes may create a platform for more enriched community engagement and immersion. Homestays also exhibit the potential to be a teaching tool, especially for improving skills that are important for medical professionals. Future research with a focus on the evaluation of homestay outcomes to medical education is urgently needed, and the potential of homestays as a component of the medical curriculum, as it shifts towards a community-based primary health-care focus, should be systematically evaluated.

## Availability of data and materials

The data supporting the conclusions of this article are accessible through the detailed reference list. No original datasets are presented, due to the fact this is a review of existing literature.
